# 2,2′-(4,6-Dinitro-1,3-phenyl­enedi­oxy)diacetic acid dihydrate

**DOI:** 10.1107/S1600536809041397

**Published:** 2009-10-17

**Authors:** Dong-Sheng Ma, Xiu-Mei Zhang, Dan Mu, Guang-Feng Hou

**Affiliations:** a College of Chemistry and Materials Science, Heilongjiang University, Harbin 150080, People’s Republic of China

## Abstract

In the title compound, C_10_H_8_N_2_O_10_·2H_2_O, the skeleton of the dicarboxylic acid mol­ecule is approximately planar, the largest deviation being 0.477 (1) Å for an O atom of a nitro group; this nitro group is twisted out of the plane of the ring by 24.6 (1)°. Adjacent mol­ecules are linked by O—H⋯O hydrogen bonds, which connect the dicarboxylic acid and water mol­ecules into a three-dimensional supra­molecular network.

## Related literature

For general background to flexible aromatic carboxylic acids, see: Coronado *et al.* (2000 ▶). For the synthesis and related structures, see: Gao *et al.* (2006 ▶) Ma *et al.* (2009 ▶)
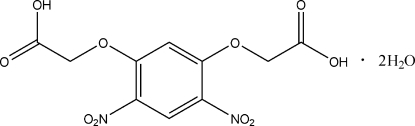


## Experimental

### 

#### Crystal data

C_10_H_8_N_2_O_10_·2H_2_O
*M* *_r_* = 352.22
Monoclinic, 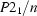

*a* = 8.4438 (17) Å
*b* = 16.049 (3) Å
*c* = 10.539 (2) Å
β = 93.13 (3)°
*V* = 1426.0 (5) Å^3^
*Z* = 4
Mo *K*α radiation
μ = 0.16 mm^−1^
*T* = 291 K
0.19 × 0.18 × 0.17 mm

#### Data collection

Rigaku R-AXIS RAPID diffractometer
Absorption correction: multi-scan (*ABSCOR*; Higashi, 1995 ▶) *T*_min_ = 0.972, *T*_max_ = 0.974
10898 measured reflections
2461 independent reflections
2059 reflections with *I* > 2σ(*I*)
*R*_int_ = 0.021

#### Refinement

*R*[*F* ^2^ > 2σ(*F* ^2^)] = 0.045
*wR*(*F* ^2^) = 0.130
*S* = 1.06
2461 reflections
217 parameters
H-atom parameters constrained
Δρ_max_ = 0.60 e Å^−3^
Δρ_min_ = −0.25 e Å^−3^

### 

Data collection: *RAPID-AUTO* (Rigaku, 1998 ▶); cell refinement: *RAPID-AUTO*; data reduction: *CrystalClear* (Rigaku/MSC, 2002 ▶); program(s) used to solve structure: *SHELXS97* (Sheldrick, 2008 ▶); program(s) used to refine structure: *SHELXL97* (Sheldrick, 2008 ▶); molecular graphics: *SHELXTL* (Sheldrick, 2008 ▶); software used to prepare material for publication: *SHELXL97*.

## Supplementary Material

Crystal structure: contains datablocks I, global. DOI: 10.1107/S1600536809041397/ng2661sup1.cif

Structure factors: contains datablocks I. DOI: 10.1107/S1600536809041397/ng2661Isup2.hkl

Additional supplementary materials:  crystallographic information; 3D view; checkCIF report

## Figures and Tables

**Table 1:** Hydrogen-bond geometry (Å, °)

*D*—H⋯*A*	*D*—H	H⋯*A*	*D*⋯*A*	*D*—H⋯*A*
O2—H10⋯O12	0.85	1.79	2.569 (2)	152
O6—H6⋯O11	0.85	1.75	2.592 (2)	169
O12—H11⋯O3^i^	0.85	1.86	2.707 (2)	171
O12—H12⋯O5^ii^	0.85	2.00	2.775 (2)	152
O11—H15⋯O10^iii^	0.85	2.04	2.852 (2)	160
O11—H14⋯O7^iv^	0.85	2.25	3.017 (3)	151
O11—H14⋯O9^ii^	0.85	2.38	2.925 (2)	123

Symmetry codes: (i) 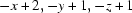; (ii) 
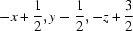
; (iii) 
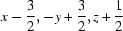
; (iv) 
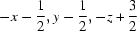
.

**Fig. 1.**
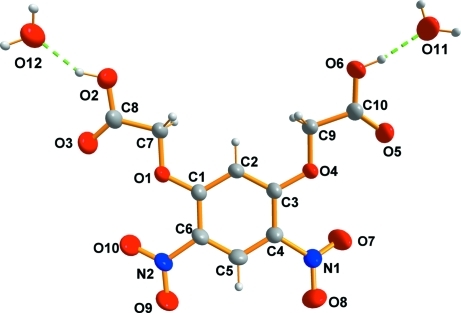
The molecular structure of the title compound, showing displacement ellipsoids at the 50% probability level for non-H atoms.

**Fig. 2.**
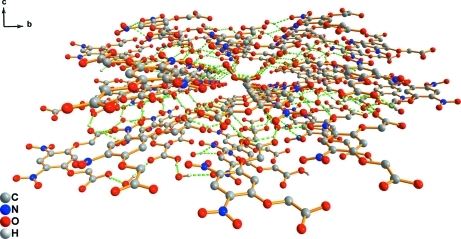
A partial packing view, showing the three-dimensional supramolecular network. Dashed lines indicate the hydrogen-bonding interactions and no involving H atoms have been omitted.

## supplementary crystallographic information

### Comment

Flexible aromatic carboxylic acid with oxygen is a kind of biological activity of the organic carboxylic acid, not only in agriculture, such as plant growth regulators and herbicides it is applied, but also it is important to the synthesis of some organic medicine centre body. Compared with other rigid carboxylic acid ligands, such flexible aromatic carboxylic acid have highly plasticity and spatial configuration of, so it provides a rich and colorful way to identify and assemble for constructing a novel topological network structure with the special physical and chemical properties (Coronado *et al.*, 2000; Gao *et al.*, 2006). In this paper, we report the synthesis and crystal structures of a new flexible aromatic carboxylic acid compound.

In the crystal structure, the skeletons of the dicarboxylic acid molecule is approximately co-planar with the largest deviation being 0.477 (1) Å from O7 of one twisty nitro group [dihedral angles = 24.6 (1)°] (Figure 1).

There are six symmetry-independent 'active' H atoms in the crystal structure, all of them participate in hydrogen bonds, which link the dicarboxylic acid and water molecules into a three-dimensional supramolecular network (Figure 2, Table 1).

### Experimental

The synthesis of target product is as follows: chlorine acetic acid (51.6 g, 0.54 mol), sodalye (36.3 g, 0.90 mol) and resorcinol (20 g, 0.18 mol) were dissolved into 200 ml distilled water with stirring. The mixture was heated to refluxed for 6 h, then the pH value was adjusted to about 2.0 by using 3 M hydrochloric acid. After cooling to the room temperature, 10.8 g (27%) yellow precipitate was obtained. The 10.8 g above yellow product was dissolved into 100 ml concentrated sulfuric acid with stirring, and then the mixture of nitric acid (9.45 g, 0.15 mol) and sulfuric acid (20.58 g, 0.21 mol) was dropped into the above solution with keeping the reaction temperature under 0° C for 1 h. Then the rection solution is stirred about 5h at room temperature. The mixture was poured into 500 ml water solution, the crude product were obtained (37%). The product was suitable for X-ray test obtained by recrystallization from water solution.

### Refinement

H atoms bound to C atoms were placed in calculated positions and treated as riding on their parent atoms, with C—H = 0.93 Å (aromatic), C—H = 0.97 Å (methylene), and with *U*_iso_(H) = 1.2*U*_eq_(C). Water and Carboxylic H atoms were initially located in a difference Fourier map, but they were treated as riding on their parent atoms with O—H = 0.85 Å and with with *U*_iso_(H) = 1.5 *U*_eq_(O).

### Crystal data

**Table:**

C_10_H_8_N_2_O_10_·2H_2_O	*F*(000) = 728
*M**_r_* = 352.22	*D*_x_ = 1.641 Mg m^−^^3^
Monoclinic, *P*2_1_/*n*	Mo *K*α radiation, λ = 0.71073 Å
Hall symbol: -P 2yn	Cell parameters from 10730 reflections
*a* = 8.4438 (17) Å	θ = 3.0–27.6°
*b* = 16.049 (3) Å	µ = 0.16 mm^−^^1^
*c* = 10.539 (2) Å	*T* = 291 K
β = 93.13 (3)°	Block, colorless
*V* = 1426.0 (5) Å^3^	0.19 × 0.18 × 0.17 mm
*Z* = 4	

### Data collection

**Table:**

Rigaku R-AXIS RAPID diffractometer	2461 independent reflections
Radiation source: fine-focus sealed tube	2059 reflections with *I* > 2σ(*I*)
graphite	*R*_int_ = 0.021
ω scans	θ_max_ = 25.0°, θ_min_ = 3.0°
Absorption correction: multi-scan (*ABSCOR*; Higashi, 1995)	*h* = −10→10
*T*_min_ = 0.972, *T*_max_ = 0.974	*k* = −18→19
10898 measured reflections	*l* = −12→12

### Refinement

**Table:**

Refinement on *F*^2^	Primary atom site location: structure-invariant direct methods
Least-squares matrix: full	Secondary atom site location: difference Fourier map
*R*[*F*^2^ > 2σ(*F*^2^)] = 0.045	Hydrogen site location: inferred from neighbouring sites
*wR*(*F*^2^) = 0.130	H-atom parameters constrained
*S* = 1.06	*w* = 1/[σ^2^(*F*_o_^2^) + (0.0711*P*)^2^ + 0.5856*P*] where *P* = (*F*_o_^2^ + 2*F*_c_^2^)/3
2461 reflections	(Δ/σ)_max_ < 0.001
217 parameters	Δρ_max_ = 0.60 e Å^−^^3^
0 restraints	Δρ_min_ = −0.25 e Å^−^^3^

### Special details

**Table:**

Geometry. All e.s.d.'s (except the e.s.d. in the dihedral angle between two l.s. planes) are estimated using the full covariance matrix. The cell e.s.d.'s are taken into account individually in the estimation of e.s.d.'s in distances, angles and torsion angles; correlations between e.s.d.'s in cell parameters are only used when they are defined by crystal symmetry. An approximate (isotropic) treatment of cell e.s.d.'s is used for estimating e.s.d.'s involving l.s. planes.
Refinement. Refinement of *F*^2^ against ALL reflections. The weighted *R*-factor *wR* and goodness of fit *S* are based on *F*^2^, conventional *R*-factors *R* are based on *F*, with *F* set to zero for negative *F*^2^. The threshold expression of *F*^2^ > σ(*F*^2^) is used only for calculating *R*-factors(gt) *etc*. and is not relevant to the choice of reflections for refinement. *R*-factors based on *F*^2^ are statistically about twice as large as those based on *F*, and *R*- factors based on ALL data will be even larger.

### Fractional atomic coordinates and isotropic or equivalent isotropic displacement parameters (Å^2^)

**Table:**

	*x*	*y*	*z*	*U*_iso_*/*U*_eq_	
C1	0.5107 (2)	0.77890 (11)	0.61383 (18)	0.0337 (4)	
C2	0.3605 (2)	0.76229 (11)	0.65578 (19)	0.0361 (4)	
H2	0.3261	0.7073	0.6589	0.043*	
C3	0.2606 (2)	0.82504 (11)	0.69305 (17)	0.0329 (4)	
C4	0.3158 (2)	0.90822 (12)	0.68891 (18)	0.0350 (4)	
C5	0.4650 (2)	0.92555 (11)	0.65097 (18)	0.0362 (4)	
H5	0.5007	0.9804	0.6511	0.043*	
C6	0.5625 (2)	0.86256 (12)	0.61261 (17)	0.0347 (4)	
C7	0.5601 (2)	0.63511 (12)	0.5756 (2)	0.0411 (5)	
H7A	0.4729	0.6256	0.5135	0.049*	
H7B	0.5256	0.6202	0.6589	0.049*	
C8	0.7010 (2)	0.58376 (13)	0.54410 (19)	0.0403 (5)	
C9	0.0654 (2)	0.72769 (11)	0.7455 (2)	0.0394 (5)	
H9A	0.1410	0.6975	0.8007	0.047*	
H9B	0.0592	0.7006	0.6631	0.047*	
C10	−0.0949 (2)	0.72744 (12)	0.80110 (18)	0.0356 (4)	
N1	0.2172 (2)	0.97868 (10)	0.72076 (18)	0.0455 (4)	
N2	0.7145 (2)	0.88676 (11)	0.56748 (17)	0.0419 (4)	
O1	0.60775 (16)	0.72016 (8)	0.57395 (15)	0.0444 (4)	
O2	0.6797 (2)	0.50642 (10)	0.5751 (2)	0.0691 (6)	
H10	0.7650	0.4796	0.5655	0.104*	
O3	0.8147 (2)	0.61073 (11)	0.4960 (2)	0.0658 (5)	
O4	0.11529 (16)	0.81155 (8)	0.73285 (14)	0.0411 (4)	
O5	−0.17145 (17)	0.78783 (9)	0.82313 (16)	0.0507 (4)	
O6	−0.13690 (18)	0.65015 (9)	0.82352 (16)	0.0517 (4)	
H6	−0.2275	0.6472	0.8546	0.077*	
O7	0.0758 (2)	0.97067 (11)	0.7144 (3)	0.0895 (8)	
O8	0.2816 (2)	1.04435 (10)	0.7480 (2)	0.0735 (6)	
O9	0.7504 (2)	0.96008 (10)	0.5705 (2)	0.0785 (6)	
O10	0.8007 (2)	0.83474 (11)	0.5236 (2)	0.0708 (6)	
O12	0.8696 (2)	0.38512 (11)	0.53639 (19)	0.0681 (5)	
H11	0.9690	0.3912	0.5312	0.102*	
H12	0.8403	0.3486	0.5886	0.102*	
O11	−0.41411 (19)	0.62047 (11)	0.90755 (19)	0.0658 (5)	
H15	−0.4880	0.6455	0.9437	0.099*	
H14	−0.4372	0.5696	0.8938	0.099*	

### Atomic displacement parameters (Å^2^)

**Table:**

	*U*^11^	*U*^22^	*U*^33^	*U*^12^	*U*^13^	*U*^23^
C1	0.0292 (10)	0.0329 (10)	0.0396 (10)	−0.0014 (7)	0.0066 (7)	−0.0016 (8)
C2	0.0316 (10)	0.0276 (9)	0.0500 (11)	−0.0041 (7)	0.0097 (8)	−0.0008 (8)
C3	0.0278 (9)	0.0306 (9)	0.0406 (9)	−0.0009 (7)	0.0058 (7)	0.0007 (8)
C4	0.0348 (10)	0.0284 (9)	0.0421 (10)	0.0001 (7)	0.0048 (8)	0.0011 (8)
C5	0.0387 (11)	0.0282 (9)	0.0419 (10)	−0.0061 (8)	0.0029 (8)	0.0038 (8)
C6	0.0292 (10)	0.0357 (10)	0.0394 (10)	−0.0059 (8)	0.0037 (7)	0.0029 (8)
C7	0.0317 (10)	0.0316 (10)	0.0609 (12)	−0.0027 (8)	0.0118 (9)	−0.0036 (9)
C8	0.0323 (10)	0.0401 (11)	0.0492 (11)	−0.0007 (8)	0.0087 (8)	−0.0063 (9)
C9	0.0355 (11)	0.0279 (9)	0.0563 (12)	−0.0013 (8)	0.0160 (9)	−0.0007 (8)
C10	0.0315 (10)	0.0360 (10)	0.0398 (10)	−0.0018 (8)	0.0067 (8)	−0.0027 (8)
N1	0.0459 (11)	0.0298 (9)	0.0621 (11)	0.0026 (7)	0.0157 (8)	0.0040 (8)
N2	0.0323 (9)	0.0402 (10)	0.0539 (10)	−0.0082 (7)	0.0071 (7)	0.0044 (8)
O1	0.0322 (7)	0.0332 (7)	0.0699 (10)	−0.0040 (6)	0.0209 (7)	−0.0073 (7)
O2	0.0519 (10)	0.0419 (9)	0.1163 (15)	0.0113 (7)	0.0309 (10)	0.0068 (9)
O3	0.0457 (10)	0.0585 (10)	0.0968 (13)	0.0050 (8)	0.0355 (9)	0.0015 (9)
O4	0.0300 (7)	0.0279 (7)	0.0669 (9)	−0.0009 (5)	0.0168 (6)	0.0000 (6)
O5	0.0364 (8)	0.0441 (8)	0.0733 (10)	0.0032 (6)	0.0183 (7)	−0.0047 (7)
O6	0.0414 (8)	0.0395 (8)	0.0765 (10)	−0.0070 (6)	0.0258 (7)	0.0029 (7)
O7	0.0444 (11)	0.0385 (9)	0.189 (2)	0.0076 (8)	0.0335 (13)	0.0009 (11)
O8	0.0688 (12)	0.0327 (9)	0.1204 (16)	−0.0043 (8)	0.0192 (11)	−0.0199 (9)
O9	0.0521 (11)	0.0445 (10)	0.1419 (19)	−0.0196 (8)	0.0315 (11)	−0.0013 (10)
O10	0.0527 (10)	0.0550 (10)	0.1092 (15)	−0.0120 (8)	0.0448 (10)	−0.0093 (10)
O12	0.0514 (10)	0.0634 (11)	0.0912 (13)	0.0040 (8)	0.0191 (9)	0.0121 (9)
O11	0.0462 (10)	0.0549 (10)	0.0995 (14)	−0.0074 (8)	0.0323 (9)	−0.0081 (9)

### Geometric parameters (Å, °)

**Table:**

C1—O1	1.332 (2)	C8—O2	1.298 (3)
C1—C2	1.392 (3)	C9—O4	1.419 (2)
C1—C6	1.412 (3)	C9—C10	1.504 (3)
C2—C3	1.384 (3)	C9—H9A	0.9700
C2—H2	0.9300	C9—H9B	0.9700
C3—O4	1.336 (2)	C10—O5	1.195 (2)
C3—C4	1.415 (3)	C10—O6	1.315 (2)
C4—C5	1.371 (3)	N1—O7	1.200 (2)
C4—N1	1.454 (3)	N1—O8	1.213 (2)
C5—C6	1.379 (3)	N2—O9	1.215 (2)
C5—H5	0.9300	N2—O10	1.216 (2)
C6—N2	1.446 (2)	O2—H10	0.8500
C7—O1	1.423 (2)	O6—H6	0.8500
C7—C8	1.499 (3)	O12—H11	0.8500
C7—H7A	0.9700	O12—H12	0.8500
C7—H7B	0.9700	O11—H15	0.8500
C8—O3	1.191 (3)	O11—H14	0.8500
			
O1—C1—C2	123.49 (17)	O3—C8—O2	125.5 (2)
O1—C1—C6	118.27 (16)	O3—C8—C7	124.2 (2)
C2—C1—C6	118.24 (17)	O2—C8—C7	110.32 (17)
C3—C2—C1	122.07 (17)	O4—C9—C10	108.50 (15)
C3—C2—H2	119.0	O4—C9—H9A	110.0
C1—C2—H2	119.0	C10—C9—H9A	110.0
O4—C3—C2	123.74 (16)	O4—C9—H9B	110.0
O4—C3—C4	118.18 (16)	C10—C9—H9B	110.0
C2—C3—C4	118.08 (17)	H9A—C9—H9B	108.4
C5—C4—C3	120.65 (17)	O5—C10—O6	125.20 (18)
C5—C4—N1	117.14 (17)	O5—C10—C9	125.55 (18)
C3—C4—N1	122.19 (17)	O6—C10—C9	109.24 (16)
C4—C5—C6	120.67 (17)	O7—N1—O8	122.56 (19)
C4—C5—H5	119.7	O7—N1—C4	118.99 (18)
C6—C5—H5	119.7	O8—N1—C4	118.39 (18)
C5—C6—C1	120.27 (17)	O9—N2—O10	121.43 (18)
C5—C6—N2	117.05 (17)	O9—N2—C6	118.43 (18)
C1—C6—N2	122.64 (17)	O10—N2—C6	120.08 (17)
O1—C7—C8	107.23 (15)	C1—O1—C7	119.71 (15)
O1—C7—H7A	110.3	C8—O2—H10	109.0
C8—C7—H7A	110.3	C3—O4—C9	117.76 (14)
O1—C7—H7B	110.3	C10—O6—H6	112.3
C8—C7—H7B	110.3	H11—O12—H12	116.4
H7A—C7—H7B	108.5	H15—O11—H14	111.3

### Hydrogen-bond geometry (Å, °)

**Table:**

*D*—H···*A*	*D*—H	H···*A*	*D*···*A*	*D*—H···*A*
O2—H10···O12	0.85	1.79	2.569 (2)	152
O6—H6···O11	0.85	1.75	2.592 (2)	169
O12—H11···O3^i^	0.85	1.86	2.707 (2)	171
O12—H12···O5^ii^	0.85	2.00	2.775 (2)	152
O11—H15···O10^iii^	0.85	2.04	2.852 (2)	160
O11—H14···O7^iv^	0.85	2.25	3.017 (3)	151
O11—H14···O9^ii^	0.85	2.38	2.925 (2)	123

Symmetry codes: (i) −*x*+2, −*y*+1, −*z*+1; (ii) −*x*+1/2, *y*−1/2, −*z*+3/2; (iii) *x*−3/2, −*y*+3/2, *z*+1/2; (iv) −*x*−1/2, *y*−1/2, −*z*+3/2.
